# Genomic Basis of Occurrence of Cryptic Resistance among Oxacillin- and Cefoxitin-Susceptible *mecA*-Positive Staphylococcus aureus

**DOI:** 10.1128/spectrum.00291-22

**Published:** 2022-05-24

**Authors:** Bingshao Liang, Zhile Xiong, Zhuwei Liang, Chao Zhang, Hao Cai, Yan Long, Fei Gao, Jielin Wang, Qiulian Deng, Huamin Zhong, Yongqiang Xie, Lianfen Huang, Sitang Gong, Zhenwen Zhou

**Affiliations:** a Clinical Laboratory, Guangzhou Women and Children’s Medical Center, Guangzhou Medical University, Guangzhou, People’s Republic of China; b Department of Gastroenterology, Guangzhou Women and Children’s Medical Center, Guangzhou Medical University, Guangzhou, People’s Republic of China; c Clinical Laboratory, Longgang District Maternity and Child Healthcare Hospital, Shenzhen, People’s Republic of China; University of West London

**Keywords:** methicillin–resistant *Staphylococcus aureus*, oxacillin– and cefoxitin–susceptible *mecA*–positive *Staphylococcus aureus*, *mecA*, *blaZ*, third-generation sequencing, quantitative polymerase chain reaction, mupirocin, oxacillin, cefoxitin, adaptive mutations, tandem repeat sequences, promoter sequence

## Abstract

The oxacillin– and cefoxitin-susceptible *mecA*–positive Staphylococcus aureus is a novel “stealth” methicillin-resistant S. aureus (MRSA) type. Here, we sequenced the whole genome of two oxacillin- and cefoxitin-susceptible *mecA*-positive MRSA isolates from breast abscesses in a lactating woman and a nasal swab of a healthy student in Guangzhou for investigating the mechanism underlying its occurrence. The reversion of these isolates was selected by exposure to sub-MICs of cefoxitin with or without mupirocin. The *mecA* expression of both parental strains and their revertants was determined, and the whole genome of the revertants was sequenced. Comparative whole-genome analyses performed for both strains revealed that *mecA* of the clinical strain was mutated by a single-bp insertion at the 262nd position in the tandem repeat region of the gene, and this mutation that led to the formation of a premature stop codon. The colonizing strain was mutated by a novel G-to-A base substitution in the second promoter region (–35 bp) of *mecA*. The *mecA* expression level of strain 697 revertant was 37 times higher than that of the parental strain. Although the *mecA* expression level was even higher for parental strain 199 compared with that for its revertant, its cDNA sequence contained a single-bp insertion. Collectively, both the missense and single substitution mutations of the second promoter of *mecA* could render MRSA isolates as “stealth” MRSA, thereby emphasizing the importance of combining phenotype tests with *mecA* or penicillin-binding protein 2a detection for the identification of MRSA.

**IMPORTANCE** The oxacillin- and cefoxitin-susceptible *mecA*-positive Staphylococcus aureus is a novel type of “stealth” methicillin-resistant S. aureus (MRSA), which is difficult to be detected using conventional methods. To investigate the genomic basis of their occurrence, we sequenced the whole genome of two previously recovered oxacillin- and cefoxitin-susceptible *mecA*-positive MRSA isolates from breast abscesses in a lactating woman and a nasal swab of a healthy student in Guangzhou. Complete SCC*mec* structure was absent except for *mecA* in clinical isolate 199. Additionally, a novel single-base pair insertion was observed in the clinical strain, which resulted in premature termination and a frameshift mutation. The colonizing isolate 697 had a Scc-*mec*-type IVa, and the second promoter region (–35 bp) of *mecA* was mutated by a novel G-to-A base substitution. The reversion of oxacillin- and cefoxitin-susceptible *mecA*-positive S. aureus to resistant MRSA isolates was selected by exposure to subminimum inhibitory cefoxitin with or without mupirocin.

## INTRODUCTION

Staphylococcus aureus colonizes asymptomatically in approximately 30% of the human population (1) and is the leading cause of bacteremia, endocarditis, skin and soft tissue infections, bone and joint infections, and hospital-acquired infections (2). Several infectious diseases caused by S. aureus have a high mortality rate. Methicillin-resistant S. aureus (MRSA), a type of “superbug,” is resistant to all β-lactam antibiotics except for the fifth-generation cephalosporin drug, ceftaroline (3). MRSA infections usually result in increased treatment costs, morbidity, and complications. MRSA bacteremia is particularly associated with increased mortality and longer hospitalization in adult patients (4). Higher rates of treatment failure and complications have also been observed in hospitalized children with MRSA bacteremia (5).

According to the Clinical and Laboratory Standards Institute (CLSI) guidelines, isolates that are tested resistant using oxacillin MIC, cefoxitin MIC, and cefoxitin disk tests or tested positive for *mecA* or low-affinity penicillin-binding protein 2a (PBP 2a) should be reported as MRSA (6). Before 2008, resistance to only oxacillin was used for MRSA screening using phenotypic assays. However, in 2001, some isolates were first reported as susceptible to oxacillin but PCR-positive for *mecA*; this discrepancy made them easily overlooked in routine screening (7, 8). These “cryptically resistant” isolates showed heterogeneity and may evolve into more homogeneously oxacillin-resistant isolates when exposed to β-lactam antibiotics *in vitro* or during clinical treatment (9, 10). These isolates were termed “dormant MRSA” in 2003 and “oxacillin-susceptible MRSA (OS-MRSA)” in 2007 (11, 12).

Cefoxitin is a potent inducer of *mecA* and supposedly more sensitive than oxacillin for screening MRSA; therefore, in 2013, cefoxitin was recommended to replace oxacillin in MRSA detection (13). However, a novel type of OS-MRSA first found in Argentina in 2011, also known as the “stealth” MRSA isolate, exhibits susceptibility to oxacillin and cefoxitin despite carrying *mecA* (14). This strain has been frequently isolated from patients and foods worldwide (15, 16), and it is more difficult to detect and could easily be misinterpreted as methicillin-susceptible S. aureus (MSSA) using the conventional method (17). These isolates can develop resistance to oxacillin and cefoxitin when exposed to β-lactam antibiotics (15, 18).

The underlying mechanisms for this “stealth” MRSA may be complicated, as many factors are involved in the resistance to methicillin-like antibiotics in MRSA. The *mecA* gene encoding PBP 2a is a prerequisite, and the promoter sequence located upstream of the *mecA* translation start site is critical (19). The *bla* transcriptional regulatory system in SCC*mec* types IV and V isolates without the functional *mecI*-*mecR1* system is vital for MRSA resistance (20). The auxiliary genes involved in cell wall metabolism, such as factors essential for methicillin resistance (*femX*, *femA*, *femB*), influence the level of methicillin resistance (21). In our previous studies, we identified two oxacillin- and cefoxitin-susceptible MRSA isolates belonging to sequence type (ST) 88 and ST59 without SCC*mec* element and with SCC*mec* IVa, respectively (1, 22). In the present study, third-generation sequencing was used to analyze the complete genomic DNA and thereby investigate the mechanism underlying the naturally occurring “stealth” MRSA isolates.

## RESULTS

### Phenotypic and genotypic characteristics of two oxacillin- and cefoxitin-susceptible *mecA*-positive MRSA isolates.

The two oxacillin- and cefoxitin-susceptible *mecA*-positive MRSA isolates in this study were isolated from breast abscesses in a lactating woman and a nasal swab of a healthy student in Guangzhou, respectively. The isolates represented two different spa types: multilocus STs and SCC*mec* types using PacBio third generation sequencing (Table 1). When isolated, they were found to be oxacillin- and cefoxitin-susceptible (using the automated VITEK2 compact system and disk diffusion tests) and were confirmed *mecA*-positive using PCR. Using MIC testing and disk diffusion tests, the nasal colonizing strain (697) was found susceptible to penicillin despite it carrying the *blaZ* gene. The *blaZ* gene encoding β-lactamase was weakly positive in the nitrocefin-based test and positive in the penicillin disk diffusion zone-edge test. The clinical strain 199 was resistant to penicillin and positive in the *blaZ* gene and β-lactamase tests (Table 1).

**TABLE 1:** The characteristics of two oxacillin- and cefoxitin-susceptible *mecA*-positive MRSA isolates and their MRSA revertants^*a*^

Isolate	Source	Origin	Spa-type	Scc*mec-*type	MLST-type	*mecA*	FOX screen	FOX DD	OXA MIC^*c*^	OXA INTPN^*d*^	PEN MIC^*c*^	PEN INTPN^*d*^	PEN DD	β-lactamase nitrocefin	β-lactamase zone-edge
199	SSTI^*a*^	Guangzhou	t17757	none	ST88	+	–	25	0.5	S	≥0.5	R	14	+	+
697	Nares	Guangzhou	t437	IVa	ST59	+	–	22	≤0.25	S	0.12	S	29	±	+
199Rna5	–	–	t17757	none	ST88	+	+	16	≥4	R	≥0.5	R	14	+	+
697R–10	–	–	t437	IVa	ST88	+	+	14	≥4	R	0.25	R	22	±	+
697R–8	–	–	t437	IVa	ST59	+	+	14	≥4	R	0.25	R	22	±	+
697R–10d6	–	–	t437	IVa	ST59	+	+	16	1	S	0.12	S	29	±	+
697R–8d6	–	–	t437	IVa	ST59	+	+	14	≥4	R	0.12	S	29	±	+
MRSA	CSF^*e*^	Guangzhou	t437	V^*b*^	ST59	+	+	16	≥4	R	≥0.5	R	16	+	+
MSSA	29213		–	–	–	–	–	28	≤0.25	S	≥0.5	R	14	+	+

a Antibiotics: FOX, cefoxitin; OXA, oxacillin; PEN, penicillin; MRSA, methicillin-resistant Staphylococcus aureus; MSSA, methicillin–susceptible S. aureus.

b SSTI, skin and soft tissue infection.

c The unit is μg/mL.

d INTPN: the interpretation was based on the guideline of the CLSI.

e Cerebrospinal fluid.

### Emergence of antibiotic resistance in two oxacillin- and cefoxitin-susceptible *mecA*-positive MRSA isolates.

**FIG 1:**
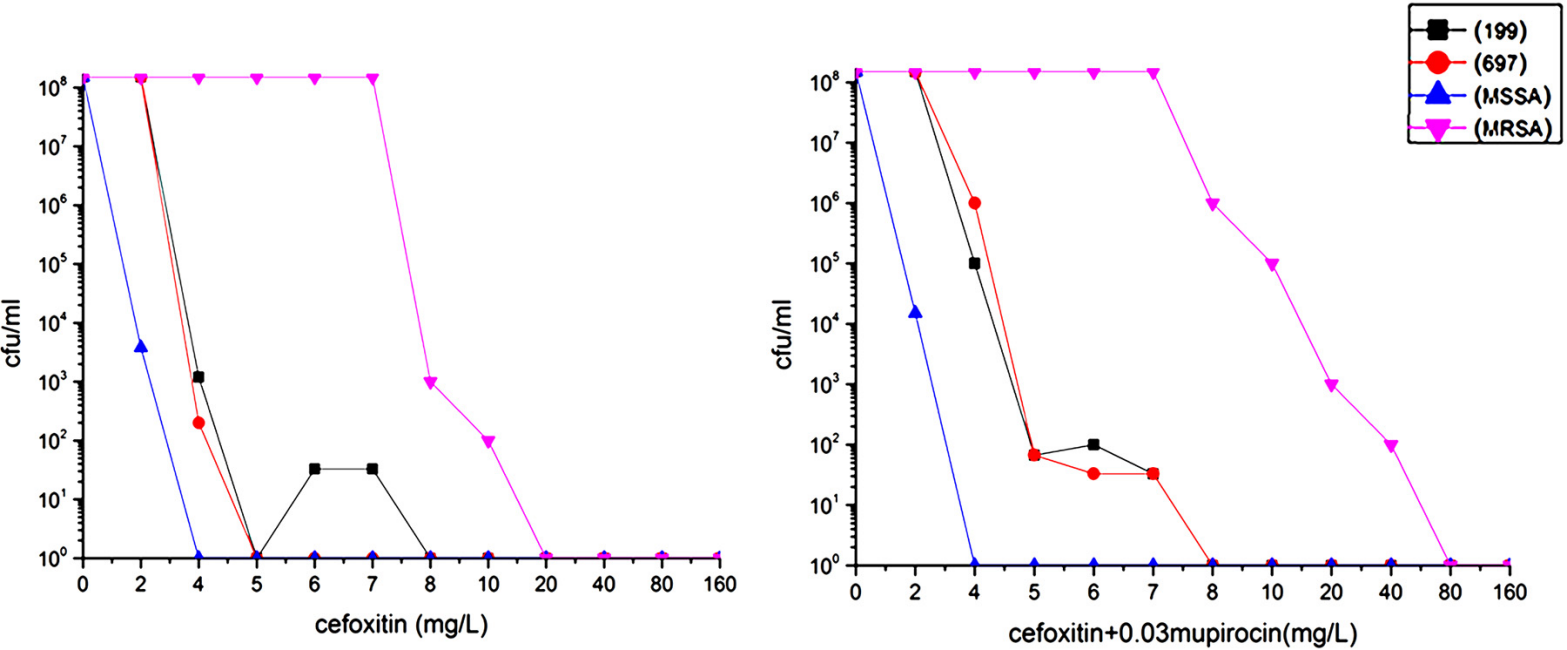
Population analysis profiles of two “stealth” MRSA parental strains (199 and 697) cultured on cefoxitin plates with or without 0.03 μg/mL mupirocin; the clinical parental strain 199 is indicated using a black line, whereas the nasal colonizing parental strain 697 using a red line. MSSA and MRSA strains were used as negative (blue line) and positive (pink line) controls, respectively. MRSA, methicillin-resistant Staphylococcus aureus; MSSA, methicillin-susceptible S. aureus.

**FIG 2:**
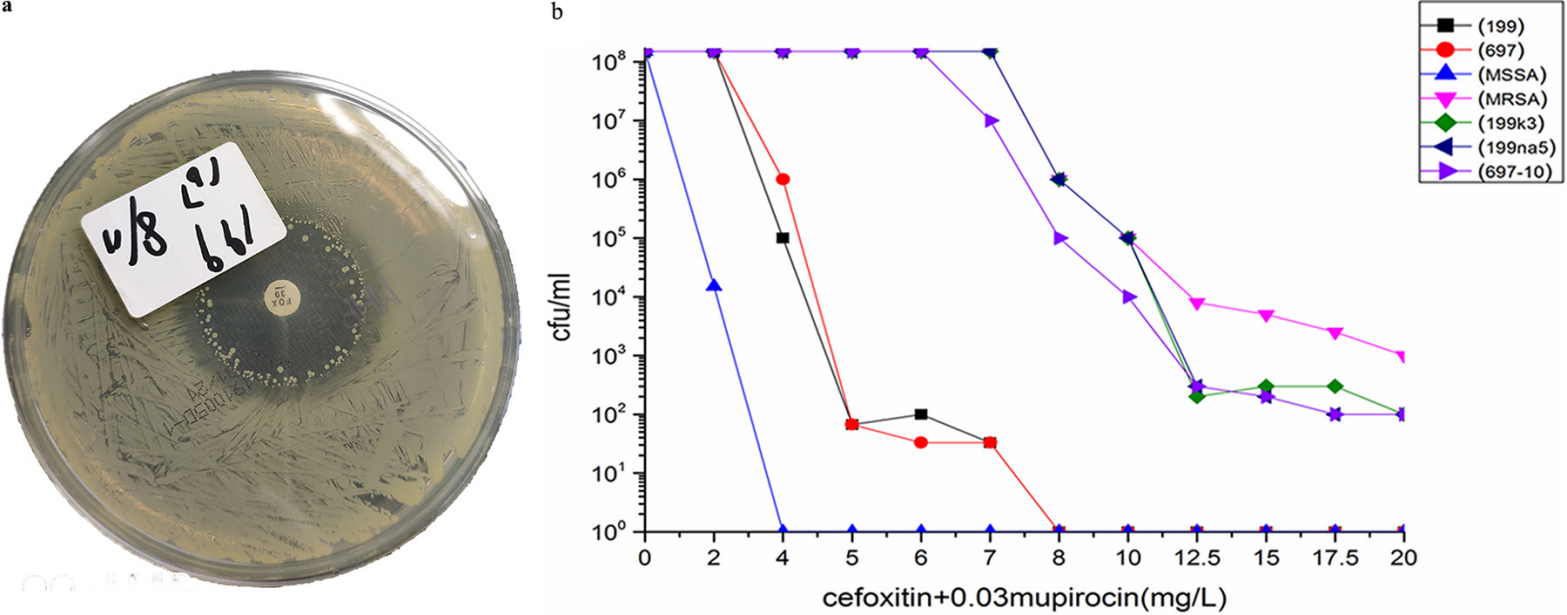
(a) Heterogeneous populations of clinical isolate 199 growing within the zones of inhibition with a higher inoculation of 10^7^ cells on a 30–μg cefoxitin disk diffusion plate incubated for 48 h. (b) The cefoxitin MIC of the 199 and 697 revertants are >20 μg/mL when cultured for 24 h on brain heart infusion agar plates containing 0.03 μg/mL mupirocin.

In the population analysis profile (PAP) tests of the two “stealth” MRSA isolates, no colonies were visible for both strains on an agar plate containing cefoxitin above 5 μg/mL after 24 h of incubation. However, some strains of the clinical isolate 199 grew and were subsequently resistant to cefoxitin and oxacillin after 48 h of incubation on sub-MIC cefoxitin plates containing 6 μg/mL and 7 μg/mL of the antibiotic. The average reversion frequency of the isolates was approximately 2.2 × 10^−7^, the average frequency of which was approximately 6.7 × 10^−7^ at 6 μg/mL cefoxitin with enhanced induction by the addition of 0.03 μg/mL mupirocin (Fig. 1). With the addition of mupirocin, the nasal colonizing isolate 697 could also grow on the sub-MIC cefoxitin plates after 48 h of incubation. The average frequency was 4.5 × 10^−7^ at 5 μg/mL cefoxitin and 2.2 × 10^−7^ at 6 and 7 μg/mL cefoxitin, although all isolates were found to be resistant to cefoxitin, oxacillin, and penicillin (Fig. 1). When 10^7^ cells were inoculated on a 30-μg cefoxitin disk diffusion plate and incubated for 48 h, the heterogeneous populations of clinical isolate 199 grew within the zones of inhibition, some of which were resistant to cefoxitin (Fig. 2a). The cefoxitin MICs of the 199 and 697 revertants were > 20 μg/mL when inoculated on brain heart infusion (BHI) agar plates containing mupirocin and cultured for 24 h (Fig. 2b). The resistant derivatives of the clinical isolate 199 were more phenotypically stable than those of the nasal colonizing isolate 697 after six generations of passages on drug-free BHI agar plates, the latter of which may be susceptible to oxacillin or penicillin, although it was resistant to cefoxitin, as suggested by the MIC screening and disk diffusion testing results (Table 1).

### Underlying mechanisms for the occurrence and reversion of two oxacillin- and cefoxitin-susceptible *mecA*-positive MRSA isolates.

**FIG 3:**
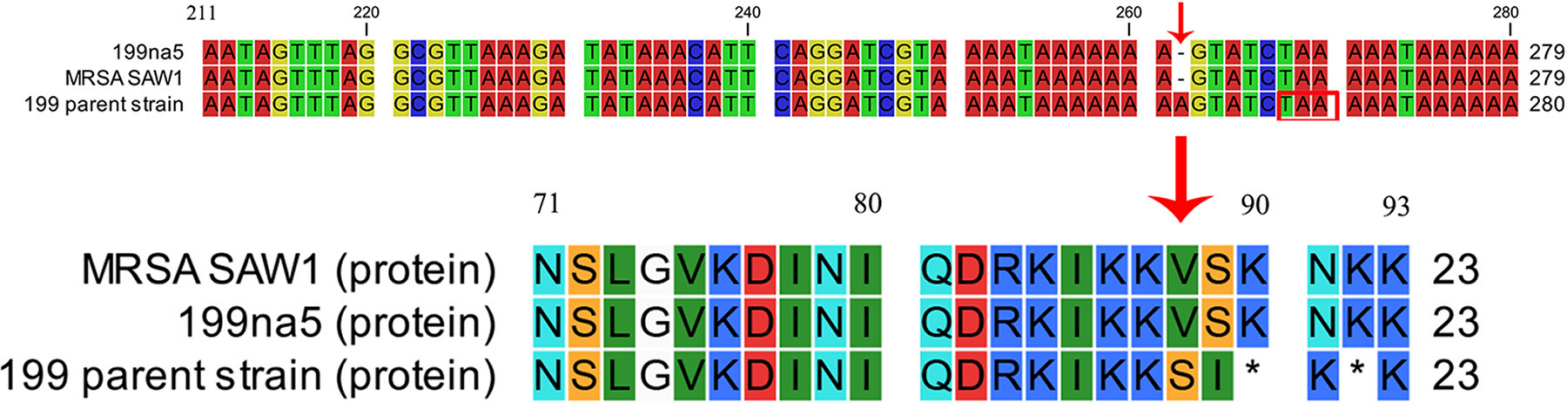
Comparison of the *mecA* gene sequence of parental strain 199 and its revertants with the *mecA* sequence of wild-type SAW1 (CP045468.1) using the CLC Genomics Workbench. The single–bp insertion at 262 bp is indicated using an arrow, and the location of a premature termination codon thereafter is marked. The diagram below specifies the corresponding amino acid sequence encoded, where, * indicates termination of peptide chain synthesis by the premature stop codon.

To investigate the genomic basis for the occurrence of two “stealth” MRSA isolates, the oxacillin- and cefoxitin-susceptible parental strains were analyzed using PacBio third-generation sequencing. Genomic analysis of clinical isolate 199 on the Center for Genomic Epidemiology website revealed the complete absence of the SCC*mec* structure except for the *mecA* gene. Rapid Annotation using Subsystem Technologies showed that *mecA* (which encodes PBP 2a) was truncated into two fragments among 37,320–39,327 bp of chromosomes and had a total length of 2,008 bp, which was one bp longer than its normal length. When aligned against the *mecA* of MRSA SAW1 strain using the CLC Genomics Workbench, a novel single-bp insertion was observed at the 262nd bp of the *mecA* gene in the tandem repeat region; this insertion resulted in a frameshift mutation and caused premature termination (Fig. 3). The mutated gene was confirmed using Sanger sequencing. No evident defects were found in other genes associated with methicillin resistance in the clinical isolate 199.

**FIG 4:**
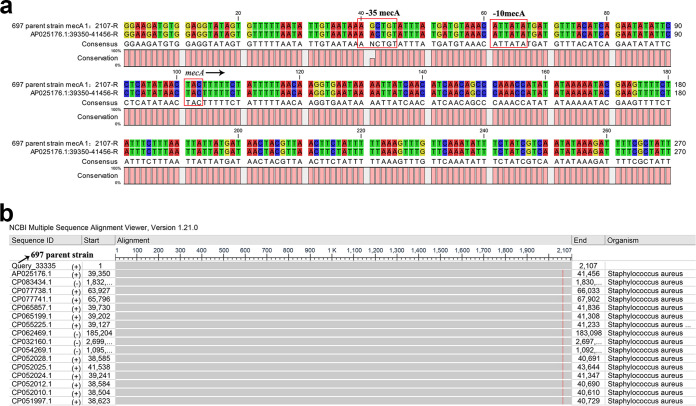
(a) Comparison of the sequences of *mecA* and its promoter region of parental strain 697 with those of TPS5614 (AP025176.1) using the CLC Genomics Workbench. The *mecA* translation start site and –10 and –35 promoter regions of the *mecA* gene are designated and labeled. (b) Comparison of the sequences of *mecA* and its promoter region of parental strain 697 using BLAST. The –35 promoter regions with the novel motif “TGTCGA” are different from those published in the NCBI database, as illustrated using the NCBI Multiple Sequence Alignment Viewer.

The complete genome of the nasal colonizing “stealth” MRSA strain 697, which was susceptible to oxacillin, cefoxitin, and penicillin, revealed that the *mecA* and *blaZ* genes were intact and identical to those of many other strains published in the National Center of Biotechnology Information (NCBI) database, carrying a Scc-*mec*-type IVa element. However, a G-to-A base substitution was observed in the 60-bp upstream region of the *mecA* translation start site, which was located in the second promoter region (–35 bp) of the *mecA* gene (Fig. 4a) (23). This is a novel mutation wherein the “TGTCGA” motif differed from all of those published in the NCBI database, as illustrated using the NCBI Multiple Sequence Alignment Viewer (Fig. 4b). Furthermore, strain 697 was partially missed by the –10 *blaZ* promoter in the plasmid, which was replaced by the “TATTGG” motif instead. The Z dyad binding site of *blaI* located in the *blaZ-blaR1* intergenic region was missed as well (23). The plasmid sequence was deposited in the NCBI database under the accession ID OL689186. Other genes that may influence the level of cefoxitin resistance were found to be truncated with premature stop codons, including *fmhC* of the *femAB*-like genes, *cstB* gene, and SCC*mec.* The genes encoding extracellular adherence protein and translocase SecA2 were also found to be mutated with premature termination.

Sanger sequencing was used to analyze the *mecA* gene sequences of all the revertants of strain 199, which were corrected by the same secondary mutations and had their reading frames restored. Third-generation sequencing method was applied to one of the resistant derivatives of strain 697; two mutated genes and 10 intergenic regions were found in the revertant compared to the genome and plasmid of the parental strain. Only the sequence of the *hisF* gene was confirmed to be different using conventional sequencing. Other mutations found in the parental strain 697 were not restored in the resistant derivative, including the –10 *blaZ* promoter sequence and the missing Z dyad binding site of *blaI*. The mutations were not reversed, although the penicillin-resistant phenotype of the revertant was resumed.

### Levels of *mecA* expression in “stealth” MRSA parental strains and revertants with cefoxitin and mupirocin inductions.

**FIG 5:**
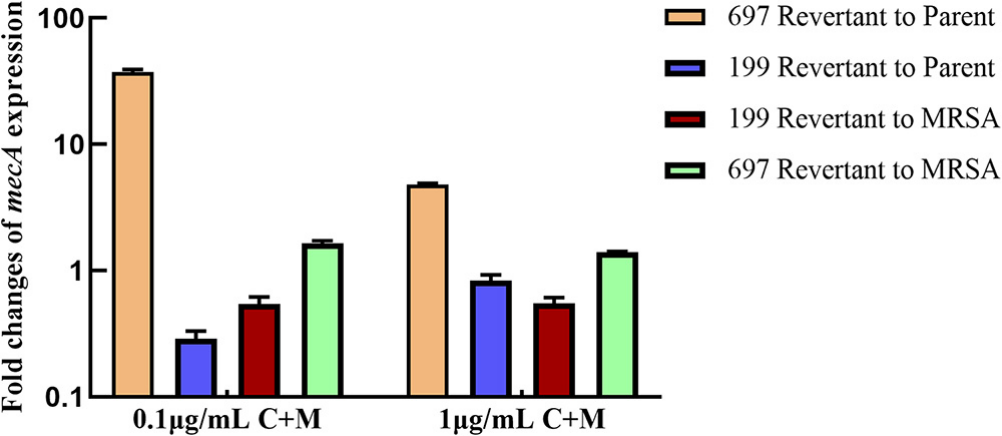
Levels of *mecA* expression in “stealth” MRSA parental strains and revertants following cefoxitin and mupirocin inductions; 0.1 μg/mL C+M and 1 μg/mL C+M indicate the antibiotic combinations 0.1 μg/mL cefoxitin + 0.03 μg/mL mupirocin and 1 μg/mL cefoxitin + 0.03 μg/mL mupirocin, respectively. Levels of *mecA* expression were compared among the “stealth” MRSA parental strains 199, 697, and their revertants in both groups as well as between the revertant and MRSA control. MRSA, methicillin-resistant Staphylococcus aureus.

The *mecA* expression levels of the parental strains and revertants were assessed using qPCR. After induction by two concentrations of cefoxitin and mupirocin for 1 h, the *mecA* expression levels of strain 697 revertant were higher than those of the parental strain, the average level of which was 37 times higher in the 0.1 μg/mL cefoxitin + 0.03 μg/mL mupirocin group and almost five times higher in the 1 μg/mL cefoxitin + 0.03 μg/mL mupirocin group. Compared with that of MRSA control, the *mecA* expression level of strain 697 revertant was higher, while that of strain 199 revertant was slightly lower (Fig. 5). These results were consistent with those of cefoxitin MIC testing (Table 1). Although the average levels of *mecA* expression of both groups were higher for the parental strain 199 than for the revertants, their cDNA sequence was the same as their genomic DNA with a single-bp insertion.

## DISCUSSION

Since 2011, oxacillin- and cefoxitin-susceptible MRSA isolates have been detected more frequently; they could not be detected using conventional susceptibility testing and therefore posed a great challenge for diagnosing and treating MRSA infections (14, 24, 25). We identified two of these true “stealth” MRSA strains from different sources representing different genetic backgrounds. The nasal colonizing strain 697 was found susceptible to penicillin, even though it carried the *blaZ* gene—a unique phenotype that has never been reported before. The β-lactamase of this strain was weakly positive in the nitrocefin-based test and positive in the more sensitive penicillin disk diffusion zone-edge test. This suggests that routinely combining phenotypic tests with *mecA* or PBP 2a detection would enable more accurate and effective identification of MRSA.

The reversion of “stealth” MRSA strain to a high-level oxacillin- and cefoxitin-resistant strain has been reported not only *in vitro* but also within patients during antibiotic treatment (18). The reversion of oxacillin- and cefoxitin-susceptible MRSA 199 was plausibly simpler than that of the nasal colonizing strain 697 because the revertants of 199 could be selected on the sub-MIC cefoxitin plate only, and some heterogeneous populations resistant to cefoxitin grew within the zones of inhibition on the 30-μg cefoxitin disk diffusion plates. Furthermore, the resistant derivatives of 199 were more phenotypically stable than the 697 derivatives after six generations. The average reversion frequency of the clinical strain was higher overall: ~6.7 × 10^−7^ when cultured on plates containing 6 μg/mL cefoxitin with 0.03 μg/mL mupirocin, which was similar to that reported by Goering et al. (15) and Proulx et al. (18). The revertants of strain 697 were only selected under the combination of the potent inducer cefoxitin and the stringent stress response induced by mupirocin. Furthermore, extending the culturing time to 48 h and increasing the number of inoculated bacteria could be more effective for the selection of revertants.

Whole-genome sequencing is a useful tool to investigate the genomic nature of the occurrence of these true “stealth” MRSA strains (21, 26). However, combining third-generation long-read sequencing and the short-read Illumina NovaSeq PE150 platform to explore the complete genome of bacteria could be better for understanding the underlying mechanisms. In this study, the *mecA* gene of clinical strain 199 was truncated into two fragments by the insertion of a single bp at 262 bp of the *mecA* gene in the tandem repeat region, resulting in premature termination. All revertants of parental strain 199 restored the *mecA* mutation by deleting the insertion and resuming the reading frame after exposure to sub-MIC antibiotics. Although the insertion of a transposable element IS1181 of the *mecA* gene has been reported as a mutation by Proulx et al. (18), to the best of our knowledge, the insertion of a single bp in the tandem repeat region has not been reported yet.

Analyses of the complete genome of the nasal colonizing “stealth” MRSA strain 697 revealed a novel mutation with a G-to-A base substitution in the second promoter region (–35 bp) of the *mecA* gene; the “TGTCGA” motif has not been reported previously (23). Furthermore, strain 697 was partially missing the –10 *blaZ* promoter region and was replaced by the “TATTGG” motif instead; these two mutations rendered MRSA isolates susceptible to cefoxitin and penicillin, respectively (23). In addition to the key factors, other mutations, including of *fmhC* of the *femAB*-like genes and *cstB* alongside SCC*mec*, may influence the level of cefoxitin resistance of the “stealth” MRSA strain 697 (27, 28). However, upon comparing the complete genome of the parental strain 697 with one of the resistant derivatives, only *hisF* gene, which is related to the synthesis of histidine, was confirmed to be different. The *hisF* gene of the resistant derivative had undergone a missense mutation, and other mutations found in the parental strain 697 were not corrected. Sequencing of the *hisF* gene, among other resistant derivatives of the parental strain 697, revealed that only two revertants carried this mutation. Therefore, we speculated that this mutation may not be the key factor involved in antibiotic reversion.

The *mecA* expression levels of strain 697 revertant were higher than those of the parental strain in both groups; the *mecA* expression level can be 37 times higher than that of the parental strain. Moreover, the *mecA* expression levels of strain 697 revertant were slightly higher than those of MRSA control, indicating that the strain 697 revertant resumed its *mecA* expressing ability and bypassed the obstacle of the *mecA* gene promoter mutation. In addition, the resistant derivatives of nasal colonizing isolate 697 were less phenotypically stable after six generations, although some of them may again become susceptible to oxacillin or penicillin, suggesting that the revertants are dependent on the strong induction of the *mecA* regulatory system by cefoxitin. The induction of strain 697 was dependent on the presence of sub-MIC cefoxitin and mupirocin, suggesting the involvement of bacterial stringent stress responses for the induction of this strain. The stringent response to environmental stress characterized by the synthesis of the messenger molecule (p)ppGpp is involved in the β-lactam resistance of MRSA (29, 30); this response can induce the conversion of a heterogeneous MRSA strain to a homogeneous phenotype exhibiting high levels of antibiotic resistance (31, 32). Collectively, our results demonstrate that missense mutations in the tandem repeat regions and substitution mutations in the second promoter region of *mecA* can enable MRSA isolates to become “stealth.” Both strains could revert to highly oxacillin- and cefoxitin-resistant phenotypes when exposed to sub-MIC antibiotics, emphasizing the importance of combining phenotype tests with *mecA* or PBP 2a detection for the identification of MRSA.

## MATERIALS AND METHODS

### Bacterial strains.

The oxacillin- and cefoxitin-susceptible *mecA*-positive MRSA clinical isolates and the nasal colonizing isolate were obtained from the clinical laboratory of Guangzhou Women and Children’s Medical Center. The characteristics of the isolates and their MRSA revertants are summarized in Table 1. The Ethics Committee of the Guangzhou Women and Children’s Medical Center approved the study protocol (registration no. 2016081029). The STs were reported in our previous studies (1, 22), and the spa-typing and SCC*mec* typing were determined using the GCE website (http://www.genomicepidemiology.org) from sequenced genomes (33). The antibiotic susceptibility testing of all isolates was performed using the automated VITEK2 compact system (bioMérieux SA, Marcy l’Étoile, France). The phenotypic resistance to cefoxitin and penicillin of the parental strains and their revertants was assessed using disk diffusion tests according to the CLSI guidelines (6). The production of β-lactamase was determined using both nitrocefin-based (Pang Tong Medical, Chongqing, China) and zone-edge tests, per the CLSI guidelines (6). The strains S. aureus ATCC 29213 and S. aureus ATCC 25923 were used for quality control.

### Isolation of strains resistant to antibiotics through PAPs and the disk diffusion method.

Both PAP tests and disk diffusion testing were conducted to identify antibiotic-resistant strains among the two oxacillin- and cefoxitin-susceptible *mecA*-positive MRSA isolates. The two isolates were cultured on blood agar plates (Guangzhou Detgerm Microbiological Science Co. Ltd., Guangzhou, China) overnight at 37°C under ambient air conditions. Bacterial suspensions with a 0.5 McFarland turbidity standard of 1.5 × 10^8^ CFU/mL were prepared using sterile 0.45% saline. The PAPs were determined by spreading 10 and 20 μL of suspensions onto BHI agar plates (Qingdao Hope Bio-Technology, Shandong, China) containing cefoxitin (2–160 μg/mL) with and without 0.03 μg/mL mupirocin (9); the plates were incubated at 37°C for 48 h. The growing colonies were confirmed to be resistant to cefoxitin. The positive colonies were subsequently detected using the automated VITEK2 compact system, and the frequency of variation was calculated. OriginPro 2017 was used to generate PAP graphs (OriginLab Corporation, Northampton, MA USA). The cefoxitin disk diffusion method (30 μg cefoxitin) was used to isolate revertants with a higher inoculation of 10^7^ cells instead; colonies that grew within the zone of inhibition were selected for confirmation of resistance. All the isolated revertants were serially passaged for six generations on drug-free BHI agar plates to determine stability of the resistance.

### RNA extraction.

Overnight cultures of the parental OS-MRSA strains 199 and 697 and their revertants were adjusted to 0.4 McFarland turbidity standard and diluted 1:100 in 1 mL Mueller-Hinton Broth. After culturing to the early log-phase (OD_600_ = 0.3) in a 37°C shaking incubator at 300 rpm, antibiotic combinations (0.1 μg/mL cefoxitin + 0.03 μg/mL mupirocin and 1 μg/mL cefoxitin + 0.03 μg/mL mupirocin) were added. The mixtures were incubated for 1 h and then spun down via centrifugation at 12,000 rpm for 2 min at 4°C. The pellets were resuspended in 600 μL TE buffer and lysed with 8 μL lysostaphin by incubating the mixtures at 37°C for 10 min. Total RNA was then extracted using the RNAprep pure Cell/Bacteria Kit (TIANGEN Biochemical Technology Co., Ltd., Beijing, China), and RNA concentrations and quality were determined using NanoDrop (Thermo Fisher Scientific [China] Co., Ltd., Shanghai, China).

### qPCR and further analysis of mecA expression among “stealth” MRSA parental strains and revertants.

Residual genomic DNA was completely removed by wipe Mix, and cDNA was synthesized via reverse transcription (Vazyme Biotech Co., Ltd, Nanjing, China). qPCR was performed on the CFX96 real-time PCR detection system (Bio-Rad Laboratories Co., Ltd, Hercules, CA, USA) using HiPer SYBR Premix EsTaq (Mei5 Biotechnology, Co., Ltd, Beijing, China). The *mecA* primer set was designed using primer Basic Local Alignment Search Tool (BLAST) for the region spanning 36–335 bp, and the housekeeping gene *gryB* was used as an internal standard for normalization (Table S1 in the supplemental material). The thermal cycling conditions were initial denaturation at 95°C for 2 min followed by 40 cycles of 95°C for 5 s and 60°C for 30 s. The experiment was repeated three times. Fold changes in *mecA* expression were illustrated using GraphPad Prism v8.0 (GraphPad Software Inc., San Diego, CA, USA). The *mecA* gene was amplified using the synthesized cDNA as template via conventional PCR. Products were sequenced using Sanger sequencing and the sequences were comparatively analyzed.

### Third-generation and conventional sequencing.

Total genomic DNA of these two “stealth” MRSA isolates and their revertants were extracted using the TaKaRa MiniBEST Bacteria GenomicDNA Extraction Kit (TaKaRa Bio Inc., Beijing, China). The whole genomes of wild-type “stealth” MRSA isolates and one revertant of the nasal swab strain were sequenced at the Beijing Genomics Institute using the long-reads PacBio third-generation sequencing method (Pacific Biosciences of California, Inc., Menlo Park, CA, USA) and the short-reads Illumina NovaSeq PE150 platform (Illumina, San Diego, CA, USA). After filtering low-quality reads, the clean data were obtained, which were preliminarily assembled using Link v5.0.1 and subsequently corrected with the Illumina data. Then, Rapid Annotation using the Subsystem Technology v2.0 server was used for gene annotation. Using BLAST, comparative genome analysis was performed among the parental and variant strains. The mutated genes were then confirmed using Sanger sequencing, and the *mecA* gene of revertants from the clinical strain was analyzed using conventional sequencing, as previously described by us (1). The sequences were aligned and further analyzed using the Qiagen CLC Genomics Workbench (33).

### Data availability.

The whole-genome sequences of both parental strains and the revertant of strain 697 have been submitted to NCBI as a BioProject (PRJNA783074) under the accession numbers CP088157, CP088158, and CP093527. The plasmids of both parental strains have been deposited at GenBank under the accession numbers OL689185 and OL689186.
